# Entwicklung des Cannabiskonsums vom Jugend- zum jungen Erwachsenenalter sowie Risiko- und Schutzfaktoren für einen problematischen Konsum – Ergebnisse einer Längsschnittstudie

**DOI:** 10.1007/s00103-025-04043-3

**Published:** 2025-04-15

**Authors:** Ann-Katrin Job, Lina-Theresa Brieske

**Affiliations:** 1 Institut fur Psychologie, Fachbereich Klinische Psychologie II, Universität Kassel, Holländische Straße 36–38, 34127 Kassel, Deutschland; 2 Institut für Psychologie, Technische Universität Braunschweig, Braunschweig, Deutschland

**Keywords:** Cannabis, Prävalenzraten, Problematischer Konsum, Risikofaktoren, Schutzfaktoren, Cannabis, Prevalence rates, Problematic cannabis use, Risk factors, Protective factors

## Abstract

**Einleitung:**

Mit dem neuen Cannabisgesetz wurde am 01.04.2024 der Konsum von Cannabis legalisiert. Bereits zuvor war Cannabis eine der am häufigsten konsumierten Drogen. Ein problematischer Konsum birgt jedoch vielfältige gesundheitliche Risiken, besonders für Jugendliche und junge Erwachsene. Die aktuelle Studie untersucht die Entwicklung des Cannabiskonsums vom Jugend- zum jungen Erwachsenenalter sowie Risiko- und Schutzfaktoren für einen problematischen Konsum bei jungen Erwachsenen.

**Methoden:**

Die Daten stammen aus der deutschen Längsschnittstudie „Zukunft Familie“ (Start: 2001, 18-Jahres-Follow-up: 2020–2022). Die Stichprobe umfasst *N* = 278 junge Erwachsene (*M* = 22,3 Jahre). Betrachtet werden Prävalenzraten und die Entwicklung des Cannabiskonsums vom Jugend- zum jungen Erwachsenenalter. Als mögliche Risiko- und Schutzfaktoren für einen problematischen Konsum werden verschiedene kind- und familienbezogene Variablen des Kindergarten- und Jugendalters untersucht.

**Ergebnisse:**

Die Lebenszeitprävalenz für Cannabiskonsum betrug 57,6 %, die Prävalenz für problematischen Konsum 13,7 %. Junge Männer hatten ein 3,3-fach höheres Risiko für einen problematischen Konsum als junge Frauen und junge Erwachsene, die bei der Befragung im Jugendalter bereits Cannabis konsumiert hatten, ein 2,6-fach höheres Risiko. Als Risikofaktor für einen problematischen Konsum erwies sich primär das Ausmaß externalisierender Verhaltensauffälligkeiten im Jugendalter aus Muttersicht.

**Diskussion:**

Die Ergebnisse bestätigen, dass Cannabiskonsum sowie ein problematischer Konsum bei jungen Erwachsenen bereits vor der Legalisierung kein seltenes Phänomen waren. Sie unterstreichen die Notwendigkeit für verhältnis- und verhaltenspräventive Maßnahmen und bieten zahlreiche Ansatzpunkte für die zukünftige Forschung.

**Zusatzmaterial online:**

Zusätzliche Informationen sind in der Online-Version dieses Artikels (10.1007/s00103-025-04043-3) enthalten.

## Abstract (en)

**Introduction:**

On April 1, 2024, the new cannabis law came into force, legalizing the use of cannabis in Germany. Even before, Cannabis had been one of the most commonly used drugs. However, problematic cannabis use is related to a variety of health risks especially for adolescents and young adults. This study examines the development of cannabis use from adolescence to young adulthood as well as risk and protective factors for problematic use among young adults.

**Methods:**

The data come from the German longitudinal study “Future Family” (Start: 2001; 18-year follow-up: 2020–22). The sample includes *N* = 278 young adults (*M* = 22.3 years). Prevalence rates and the development of cannabis use from adolescence to young adulthood are analyzed. Various child- and family-related variables from early childhood and adolescence are examined as potential risk and protective factors for problematic cannabis use.

**Results:**

The lifetime prevalence of cannabis use was 57.6%, and the prevalence of problematic use was 13.7%. Young men had a 3.3 times higher risk of problematic cannabis use compared to young women. Young adults who had already used cannabis at least once in their youth when surveyed had a 2.6 times higher risk of problematic use in young adulthood. The extent of mother-reported externalizing behavior problems in adolescence significantly predicted problematic use eight years later.

**Discussion:**

The results confirm that cannabis use and problematic use in young adulthood were not a rare phenomenon even before legalization. They highlight the need for both conditional and behavioral preventive actions and provide numerous starting points for future research.

## Einleitung

Seit dem 01.04.2024 ermöglicht das neue Cannabisgesetz (CanG) Erwachsenen den privaten Eigenanbau von Cannabis zum Eigenkonsum. Die Legalisierungsdebatte wurde bereits im Vorfeld kontrovers geführt. Diverse Fachverbände haben ihre Einschätzung zu den Chancen und Risiken der Legalisierung wiederholt in Stellungnahmen dargelegt. Studien aus anderen Ländern, wie kürzlich in den USA, zeigen, dass es nach einer Legalisierung von Cannabis in den Folgejahren zu einem signifikanten Anstieg an Cannabiskonsumstörungen (CUD) sowie Cannabisvergiftungen in der Bevölkerung kommt [1]. Vor diesem Hintergrund wurden immer wieder die mit Cannabis verbundenen gesundheitlichen Risiken betont und damit einhergehend erhebliche Bedenken bezüglich des Jugendschutzes [2–4]. Für Jugendliche und junge Erwachsene ist Cannabis besonders gefährlich, da ein früher Konsum das Risiko für die Entwicklung kognitiver Beeinträchtigungen [5] und psychischer Erkrankungen, wie Psychosen [6], Depressionen und Suizidalität [7], erhöht. Ein früher wöchentlicher bis täglicher Konsum steht darüber hinaus mit schlechteren schulischen Leistungen und einem schlechteren akademischen Erfolg in Zusammenhang [8].

In Deutschland gehört Cannabis zu den am meisten konsumierten Drogen [9]. Laut einer repräsentativen Studie der Bundeszentrale für gesundheitliche Aufklärung (BZgA; [10]) lag die Lebenszeitprävalenz (LZP) für Cannabiskonsum bei 12- bis 17-Jährigen im Jahr 2021 bei 9,3 %, die 12-Monats-Prävalenz (12‑M.-P.) bei 7,6 % und die 30-Tages-Prävalenz (30‑T.-P.) bei 3,5 %. Einen regelmäßigen Cannabiskonsum, d. h. mehr als 10-mal in den letzten 12 Monaten, gaben 1,6 % der Jugendlichen an. Bei 18- bis 25-Jährigen betrug die LZP 50,8 %, die 12‑M.-P. 25,0 %, die 30‑T.-P. 12,0 % und 8,6 % gaben an, regelmäßig Cannabis zu konsumieren. Im Hinblick auf Cannabiskonsumstörungen (CUD) lag die 12‑M.-P. in einer weiteren repräsentativen deutschen Studie mit Jugendlichen im Alter von 12–18 Jahren bei 2,6 % und unter denjenigen, die im letzten Jahr Cannabis konsumiert hatten, bei 39,7 % [11]. Laut einer Metaanalyse liegt die 12‑M.P. für CUD unter Konsument:innen über alle Altersgruppen hinweg international bei ca. 22 % [12].

Auch wenn zahlreiche Fachverbände die geplanten Maßnahmen für nicht ausreichend erachten und ihre Umsetzung infrage stellen [2–4], sieht das CanG aufgrund der Risiken vor allem für junge Menschen eine Maximierung des Kinder- und Jugendschutzes vor. Neben einer Mindestaltersgrenze von 18 Jahren für den Verkauf und Erwerb von Cannabis sollen u. a. cannabisspezifische Präventionsangebote evidenzbasiert weiterentwickelt und ausgebaut werden. Bislang gibt es wenige Studien, die den Beginn und die Entwicklung eines riskanten Cannabiskonsums systematisch untersuchen [13]. Auch fehlen Längsschnittstudien, die gleichzeitig verschiedene Risiko- und Schutzfaktoren betrachten [13, 14]. Die aktuelle Studie nutzt daher Daten einer prospektiven Längsschnittstudie über 18 Jahre, um einerseits die Entwicklung des Cannabiskonsums vom Jugend- zum jungen Erwachsenenalter zu untersuchen und andererseits personale und familiäre Prädiktoren für einen riskanten Cannabiskonsum zu identifizieren und so Ansatzpunkte für gezielte Präventionsangebote zu liefern. Der Fokus liegt dabei auf der Vorhersage eines problematischen Konsums im jungen Erwachsenenalter, da für junge Menschen mit einem riskanten Konsum das höchste Risiko für langfristige negative Folgen besteht.

Laut metaanalytischen Befunden stellen das Vorliegen einer Aufmerksamkeitsdefizit- und Hyperaktivitätsstörung (ADHS) sowie frühes antisoziales Verhalten mit Beginn in der Kindheit und Jugend signifikante Risikofaktoren für eine spätere CUD dar [15]. Damit übereinstimmend erwiesen sich in der prospektiven deutschen Mannheimer Risikokinderstudie ein gestörtes Sozial- sowie oppositionelles Verhalten in der Kindheit und Aufmerksamkeitsprobleme im Jugendalter als prädiktiv für einen problematischen Cannabiskonsum im Alter von 25 Jahren [16]. In einer retrospektiven Online-Kohortenstudie [13] mit Cannabiskonsumierenden im Alter von 18–35 Jahren wurden folgende Risikofaktoren für einen riskanten Konsum identifiziert: männliches Geschlecht, höheres Alter, ein Migrationshintergrund, höheres Sensation Seeking, früherer Erstkonsum, Cannabiskonsum im Freundeskreis während der Schulzeit, positive soziale Reaktionen auf den Konsum vor dem 16. Lebensjahr, eine instabilere Eltern-Kind-Beziehung und geringe psychische Gesundheit der Eltern. Als nicht signifikant erwiesen sich der sozioökonomische Status (SÖS), der Erziehungsstil der Eltern und das Vorhandensein eines ADHS. In weiteren Studien erwiesen sich externalisierende Verhaltensauffälligkeiten im Jugendalter [17, 18], ein regelmäßiger Konsum von Alkohol und Tabak [19], Cannabiskonsum im Jugendalter [20, 21], elterlicher Substanzkonsum [22, 23], Armut und ein niedriger SÖS [18, 23, 24] als signifikante Prädiktoren für einen späteren Cannabiskonsum bzw. die Entwicklung eines problematischen Konsums. Eine enge Eltern-Kind-Beziehung und familiäre Bindung im Jugendalter wiederum können das Risiko mindern [18, 23].

Die deutsche Längsschnittstudie *Zukunft Familie* umfasst Informationen von Eltern und ihren Kindern vom Kindergarten- bis zum jungen Erwachsenenalter. Es werden die LZP, 12‑M.-P. und 30‑T.-P. von Cannabiskonsum im jungen Erwachsenenalter sowie die Prävalenz von problematischem Konsum bestimmt und Geschlechterunterschiede betrachtet. Da ein Großteil der Befragungen im jungen Erwachsenenalter während der COVID-19-Pandemie stattfand, wird berichtet, inwiefern sich das Konsumverhalten durch die Pandemie verändert hat. Als erste Hauptfragestellung wird die Entwicklung des Cannabiskonsums vom Jugend- zum jungen Erwachsenenalter betrachtet. Anschließend wird als zweite Hauptfragestellung untersucht, welche prospektiv erfassten personalen und familiären Faktoren im Kindes- und Jugendalter das Risiko für die Entwicklung eines problematischen Konsums im jungen Erwachsenenalter beeinflussen, um Ansatzpunkte für Prävention abzuleiten. Dabei werden die folgenden aus der Literatur abgeleiteten möglichen Prädiktoren betrachtet: biologisches Geschlecht, Alter, Vorhandensein eines Migrationshintergrunds, verschiedene Indikatoren für den SÖS der Familie (z. B. Nettohaushaltseinkommen, höchster Schulabschuss der Eltern), das Ausmaß internalisierender und externalisierender Verhaltensauffälligkeiten im Kindes- und Jugendalter, die besuchte Schulform und Substanzkonsum im Jugendalter, die psychische Belastung der Eltern sowie elterlicher Substanzkonsum und die Qualität der Eltern-Kind-Beziehung im Jugendalter.

## Methode

### Studiendesign

Das Projekt „Zukunft Familie“ (ZF) startete 2001 und bestand ursprünglich aus zwei Studien, die die Wirksamkeit des präventiven Elterntrainings „Triple P“ (Positive Parenting Program; [25]) untersuchten. Bei der von der Deutschen Forschungsgemeinschaft (DFG) geförderten ZF-I-Studie (Förderkennzeichen: HA 1400/14-1‑3; 4-5) handelte es sich um eine randomisiert kontrollierte universelle Präventionsstudie, bei der *N* = 280 Familien über 17 zufällig ausgewählte Kindertagesstätten der Stadt Braunschweig rekrutiert wurden. Von diesen wurde *n* = 186 Familien die Teilnahme an einem Triple-P-Elterngruppentraining angeboten und *n* = 94 wurden der Kontrollgruppe zugeordnet. An der ZF-II-Studie, bei der es sich um eine von der Jacobs-Stiftung geförderte nichtkontrollierte selektive Präventionsstudie handelte, nahmen *N* = 197 Familien aus sozial benachteiligten Stadtgebieten in Braunschweig teil. Das Ziel dieser Studie war es zu untersuchen, ob finanzielle Anreize die Teilnahmebereitschaft am Triple P erhöhen und ob unterschiedliche Settings (Gruppe vs. Einzel) einen Einfluss auf die Wirksamkeit haben. Weitere Informationen zum Studiendesign und zur Rekrutierung finden sich bei Hahlweg und Schulz [26].

Im Rahmen von ZF-III wurden 2011–2013 das 10-Jahres-Follow-up (FU10; [26]) und von ZF-IV in den Jahren 2020–2022 das 18-Jahres-Follow-up (FU18) der ZF-I- und der ZF-II-Stichprobe erhoben. Bei ZF-III und ZF-IV, die beide ebenfalls von der DFG gefördert wurden (Förderkennzeichen ZF-III: HA 1400/17‑1,2; ZF-IV: JO 1632/1-1), umfasste die Datenerhebung jeweils ein ca. 1,5- bis 2,5-stündiges persönliches Interview und das Ausfüllen von Fragebögen für die Jugendlichen bzw. jungen Erwachsenen und Eltern. Die freiwillige Teilnahme wurde mit 40 € (ZF-III) bzw. 50 € (ZF-IV) pro Person vergütet.

### Rekrutierung und Stichprobe

Bei der ersten Erhebung im Kindergartenalter (Messzeitpunkt „Prä“) waren die Kinder im Mittel 4,1 Jahre alt (*SD* = 1,0; Range: 2,5–6). Von den insgesamt *N* = 477 Familien (280 ZF-I- und 197 ZF-II-Familien) beteiligten sich am FU10 (ZF-III) noch *N* = 361 Familien (Retentionsrate: 75,7 %), hier waren die Jugendlichen im Mittel 14,1 Jahre alt (*SD* *=* 1,2; Range: 11–17). Nach dem Ausschluss aufgrund fehlender Cannabisdaten (*n* = 2) lagen zum FU18 die Daten von *N* = 278 jungen Erwachsenen vor (Retentionsrate: 59,0 %). *N* = 6 Familien wurden nicht erneut befragt, da sie die Einschlusskriterien nicht erfüllten, und *n* = 2 junge Erwachsene waren bereits verstorben. Das mittlere Alter der jungen Erwachsenen betrug 22,3 Jahre (*SD* = 1,2; Range: 19–26), 50 % waren männlich. Weitere Angaben zur untersuchten Stichprobe sowie über die drei Messzeitpunkte finden sich in Tab. Z1 und Z2 im Onlinematerial.

#### Dropout-Analyse.

Die Familien, die am FU18 nicht teilnahmen, hatten zu Studienbeginn (Prä) häufiger einen niedrigen SÖS (höchster Schulabschluss der Eltern, Nettohaushaltseinkommen; jeweils *p* ≤ 0,001) und einen Migrationshintergrund (*p* = 0,036). Die Mütter waren zu Prä jünger (*p* ≤ 0,001), häufiger alleinerziehend (*p* ≤ 0,001) und berichteten eine höhere psychische Belastung (*p* = 0,027) sowie stärkere Verhaltensauffälligkeiten bei ihren Kindern (*p* ≤ 0,004). Die Repräsentativität der FU18-Stichprobe ist demnach gegenüber der Ursprungsstichprobe eingeschränkt.

### Messinstrumente

#### Cannabiskonsum im Jugendalter (FU10).

Anhand eines Fragebogenitems wurden die Jugendlichen gefragt, ob sie schon einmal Cannabis konsumiert haben. Jugendliche, die diese Frage mit „Ja“ beantworteten, erfüllten das Kriterium für Cannabiskonsum im Jugendalter. Weitere Antwortkategorien waren „Nein“ und „Ich kenne diese Substanz nicht“.

#### Cannabiskonsum im jungen Erwachsenenalter (FU18).

Die Konsumhäufigkeit von Cannabis bzw. Marihuana und Haschisch im jungen Erwachsenenalter wurde anhand eines 5‑stufigen Items erfragt (1 = nie bis 5 = regelmäßig). Zusätzlich wurde auf einer 6‑stufigen Skala erfasst, wann das letzte Mal eine illegale Substanz konsumiert wurde (1 = nicht in den letzten 12 Monaten bis 6 = heute), und auf einer 5‑stufigen Skala abgefragt, inwieweit sich der Konsum illegaler Drogen seit dem Beginn der COVID-19-Pandemie verändert hat (1 = viel mehr als zuvor; 5 = viel weniger als zuvor).

#### Problematischer Cannabiskonsum (FU18).

Zur Erfassung eines problematischen Cannabiskonsums wurde der „Cannabis Use Disorders Identification Test-Revised“ (CUDIT‑R [27]) eingesetzt. Die 8 Items werden zu einem Gesamtwert aufsummiert (Min = 0; Max = 32; α = 0,81). Da für Deutschland bislang keine Cut-off-Werte vorliegen, wurde auf die Empfehlung von Bonn-Miller et al. [28] zurückgegriffen, die für nichtklinische Stichproben einen Cut-off von ≥ 10 empfehlen. In ihrer Studie, in der eine US-amerikanische (*N* = 207; 24 % weiblich; Alter: *M* = 41 Jahre, *SD* = 14,8) und eine australische (*N* = 369; 37 % weiblich; Alter: *M* = 28,1 Jahre, *SD* = 10,9) Stichprobe untersucht wurden, wies dieser Cut-off die beste Kombination aus Sensitivität und Spezifität zur Identifikation einer milden CUD nach DSM‑5 auf.

Die weiteren in dieser Studie verwendeten Instrumente sind der Tab. 1 zu entnehmen.

**Tab. 1:** Übersicht über die Messinstrumente differenziert nach dem Erhebungszeitpunkt (Prä, FU10) und den untersuchten Personen (junge Erwachsene, Mütter, Väter)

Merkmal	Messinstrument	
	Prä	FU10
Soziodemografische Variablen des Kindes	In den persönlichen Interviews erhoben: biologisches Geschlecht Kind, Alter Kind	In den persönlichen Interviews erhoben: besuchte Schulform (Gymnasium vs. andere Schulform)
Soziodemografische Variablen der Eltern	In den persönlichen Interviews erhoben: Migrationshintergrund mind. eines Elternteils (Ja/Nein), monatliches Nettohaushaltseinkommen (< 3000 DM, 3000 bis < 6000 DM, > 6000 DM), höchster Schulabschluss der Eltern (ohne Abschluss/Hauptschule, mittlere Reife, Abitur)	–
Sozioökonomischer Status (SÖS)	Scheuch-Winkler-Index (niedriger/mittlerer vs. hoher SÖS; [29])	Sozioökonomischer Status (niedrig/mittel vs. hoch; [30])
Teilnahme am Triple-P-Elterntraining	Interventionsgruppe (IG) vs. Kontrollgruppe (KG)	–
Kindliche internale und externale Verhaltensauffälligkeiten, Aufmerksamkeitsprobleme	Fremdeinschätzung der Mütter: dt. Version der Child Behavior Checklist (CBCL 6‑18R; [31]), Syndromskalen internalisierende und externalisierende Verhaltensauffälligkeiten	(a) Selbsteinschätzung: dt. Version des *Youth Self-Report* (YSR 11–18; [32]) und (b) Fremdeinschätzung der Mutter: dt. Version der Child Behavior Checklist (CBCL 4–18 [32]), jeweils Syndromskalen internalisierende und externalisierende Verhaltensauffälligkeiten; Skala Aufmerksamkeitsprobleme
Elterliche psychische Belastung (Mutter, Vater)	Dt. Version der Depression Anxiety Stress-Scale (DASS 42, [33]), Gesamtwert	
Alkoholkonsum des Kindes	–	In den persönlichen Interviews erhoben: „Nein, kein Konsum oder nur einmal probiert“ vs. „Ja, mehrfach“
Tabak‑, Cannabis- und illegaler Drogenkonsum generell	–	In den persönlichen Interviews erhoben: „Nein, kein Konsum“ vs. „Ja, mind. 1 × probiert“
Illegaler Drogenkonsum im Freundeskreis (inkl. Cannabis)	–	Im Fragebogen erhoben: 0 = keiner, 1 = ein:e Freund:in, 2 = mind. 2 Freund:innen
Elterlicher Tabakkonsum Mutter/Vater	–	In den persönlichen Interviews erhoben: „Nein, kein Konsum“ vs. „Ja, gelegentlich oder täglich“
Elterliche alkoholbezogene Störung (Mutter, Vater)	–	Lübecker Alkohol Screening Test (LAST; [34]), Summenwert
Qualität der Eltern-Kind-Beziehung	–	Elternbildfragebogen für Kinder und Jugendliche (EBF-KJ; [35]), Skalen Kohäsion (5 Items), Konflikte (4 Items), Ablehnung/Gleichgültigkeit (4 Items) und Überprotektion (4 Items)

### Statistische Auswertung

Die Ermittlung der Prävalenzraten sowie die Betrachtung der Veränderung des Cannabiskonsums durch die COVID-19-Pandemie und der Konsumentwicklung vom Jugend- zum jungen Erwachsenenalter erfolgte deskriptiv, der Vergleich zwischen Männern und Frauen mittels *χ2*-Unabhängigkeitstest. Zusätzlich werden für das biologische Geschlecht und den Cannabiskonsum im Jugendalter als Maß für den jeweiligen Einfluss das Relative Risiko mit der Formel RR = (a/[a+c])/(b/[b+d]) berechnet und das 95 %-Konfidenzintervall (KI) mit angegeben. Bezüglich des Zusammenhangs der einzelnen prospektiv erfassten personalen und familiären Faktoren des Kindes- und Jugendalters mit problematischem Cannabiskonsum im jungen Erwachsenenalter wurden zwecks Variablenselektion zunächst Gruppenvergleiche mittels *χ2*-Unabhängigkeitstests und *t*-Tests berechnet. Zusätzlich wurden Effektstärken bestimmt; die Interpretation orientiert sich an den üblichen Richtwerten (klein: φ, r ≥ 0,10; mittel: φ, r ≥ 0,30; stark: φ, r ≥ 0,50). Zur Vorhersage des problematischen Konsums wurden anschließend getrennt für das Kindes- und Jugendalter mit allen Variablen, die sich in den paarweisen Vergleichen als signifikant erwiesen hatten, nonparametrische Klassifikationsbäume unter Verwendung der QUEST-Aufbaumethode (Quick, Unbiased, Efficient Statistical Tree) für nominal abhängige Variablen berechnet. Klassifikationsbäume erlauben die Aufteilung von Fällen in verschiedene Gruppen, indem sie Regeln erstellen und diese verwenden, um zukünftige Ereignisse vorherzusagen. Dieses Analyseverfahren bietet im Vergleich zu einer logistischen Regression den Vorteil, dass es nichtparametrisch ist und gleichzeitig eine unbegrenzte Menge an metrischen, ordinalen und nominalen unabhängigen Variablen verarbeiten kann [36]. Die QUEST-Aufbaumethode ermittelt in jedem Schritt diejenige unabhängige Variable, welche die größte Interaktion mit der abhängigen Variable aufweist. Aufgrund der eingeschränkten Stichprobengröße wurde die Mindestanzahl der Fälle im übergeordneten Knoten auf *n* = 10 und im untergeordneten Knoten auf *n* = 5 festgelegt. Die Analysen wurden mit IBM SPSS 28 durchgeführt [37].

## Ergebnisse

### Cannabiskonsum im jungen Erwachsenenalter

Die LZP für Cannabiskonsum lag bei 57,6 % (*n* = 160), die 12‑M.-P. bei 35,3 % (*n* = 98) und die 30‑T.-P. bei 20,1 % (*n* = 56). Einen problematischen Konsum wiesen 12,2 % (*n* = 34) der jungen Erwachsenen auf.

#### Geschlechterunterschiede.

Männliche junge Erwachsene gaben 1,30-mal so häufig an, in ihrem Leben bereits Cannabis konsumiert zu haben, als weibliche (*χ2* = 6,59; *p* = 0,005; 95 %-KI: 1,06–1,60). Weiterhin gaben sie 1,75-mal so häufig an, in den letzten 12-Monaten (*χ2* = 11,24; *p* ≤ 0,001; 95 %-KI: 1,25–2,45), und 1,98-mal so häufig in den letzten 30 Tagen (*χ2* = 7,57; *p* = 0,003; 95 %-KI: 1,20–3,26), Cannabis konsumiert zu haben. Das Risiko für einen problematischen Konsum war bei den jungen Männern um das 3,30-Fache erhöht (*χ2* = 11,16; *p* ≤ 0,001; 95 %-KI: 1,54–7,03). Die Effektstärken fielen gering aus (φ ≤ 0,20). Eine Übersicht über die geschlechtsspezifischen Prävalenzraten gibt Tabelle Z3 im Onlinematerial.

#### Einfluss der COVID-19-Pandemie.

Da die Frage zur pandemiebedingten Veränderung des Konsums erst ab September 2020 erhoben wurde, lag diese Angabe nur für *n* = 101 (63,1 %) cannabiskonsumierende junge Erwachsene vor. Von diesen gaben lediglich 4,0 % (*n* = 4) an, seit Beginn der Pandemie viel mehr, und 7,9 % (*n* = 8), etwas mehr zu konsumieren. Mehr als Dreiviertel (*n* = 77, 76,2 %) gaben keine Veränderung an und jeweils 5,9 %, etwas (*n* = 6) bzw. viel weniger (*n* = 6) zu konsumieren.

### Entwicklung des Konsums vom Jugend- zum jungen Erwachsenenalter

**Abb. 1:**
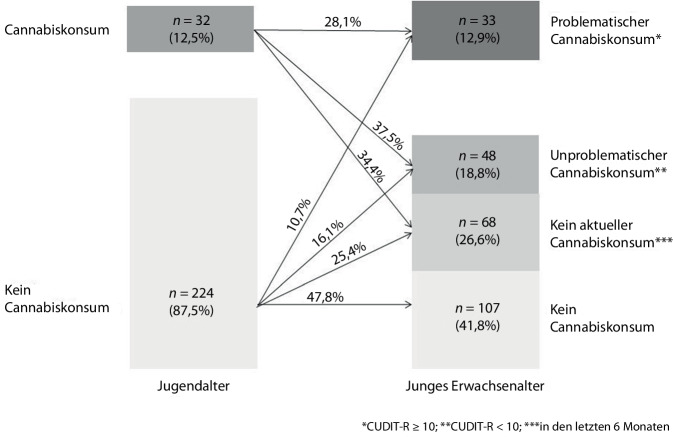
Entwicklung des Cannabiskonsums vom Jugend- zum jungen Erwachsenenalter (*N* = 256). Quelle: eigene Abbildung

Für einige junge Erwachsene lagen keine Daten zum Cannabiskonsum im Jugendalter vor, sodass hier lediglich eine Stichprobe von *n* = 256 (92,1 %) betrachtet werden konnte. Die jungen Erwachsenen, für die keine Daten vorlagen, unterschieden sich nicht signifikant von denen mit vollständigen Daten. Abb. 1 zeigt die Entwicklung des Konsums vom Jugend- zum jungen Erwachsenenalter. Im Jugendalter gaben 12,5 % (*n* = 32) der Befragten an, bereits mindestens einmal Cannabis konsumiert zu haben. Von diesen konsumierten im jungen Erwachsenenalter etwas mehr als ein Drittel unproblematisch (37,5 %; *n* = 12) und knapp ein Drittel problematisch (28,1 %; *n* = 9) Cannabis. Von den Jugendlichen, die zum FU10 noch kein Cannabis konsumiert hatten (87,5 %; *n* = 224), wiesen im jungen Erwachsenenalter lediglich 10,7 % (*n* = 24) einen problematischen Konsum auf. Das Risiko für einen problematischen Konsum, war bei einem frühen Einstieg um das 2,63-Fache erhöht (95 %-KI: 1,34–5,13).

### Vorhersage eines problematischen Konsums durch Variablen des Kindesalters

Die Ergebnisse der paarweisen Vergleiche mit den Variablen des Kindesalters (Prä) sind Tab. 2 zu entnehmen. Da sich abgesehen vom biologischen Geschlecht (*d* = 0,20; s. oben) lediglich für die Höhe des Nettohaushaltseinkommens der Familie (*p* = 0,034; *d* = 0,16) ein signifikanter Unterschied zwischen jungen Erwachsenen mit und ohne problematischen Konsum ergab, wurden nur diese 2 Variablen als mögliche Prädiktoren in die Berechnung des Klassifikationsbaums mit aufgenommen. Hierbei erwies sich ausschließlich das Geschlecht als signifikante Trennvariable.

**Tab. 2:** Paarweiser Vergleich potenzieller Prädiktoren des Kindesalters (Prä) für einen problematischen Cannabiskonsum im jungen Erwachsenenalter (*N* = 278)

		Problematischer Cannabiskonsum					
		Nein	Ja				
Variablen des Kindesalters	***N***	***n* (%)**	***n* (%)**	**χ2**	**Df**	***p* (2-seitig)**	**φ**
*Biologisches Geschlecht*				11,2	1	≤ 0,001***	0,20
– Männlich	138	112 (81)	26 (19)				
– Weiblich	140	132 (94)	8 (6)				
*Triple-P-Teilnahme*				0,8	1	0,375	0,05
– Interventionsgruppe	213	189 (89)	24 (11)				
– Kontrollgruppe	65	55 (85)	10 (15)				
*Migrationshintergrund*				2,3	1	0,133	0,09
– Ja	55	45 (82)	10 (18)				
– Nein	223	199 (89)	24 (11)				
*Monatliches Nettohaushaltseinkommen*				6,8	2	0,034*	0,16
– Unter 3000 DM	60	48 (80)	12 (20)				
– 3000 bis unter 6000 DM	137	127 (93)	10 (7)				
– > 6000 DM	73	63 (86)	10 (14)				
*Höchster Schulabschluss der Mutter*				0,7	2	0,692	0,05
– Ohne Abschluss/Hauptschule	31	26 (84)	5 (16)				
– Mittlere Reife	104	93 (89)	11 (11)				
– Abitur	141	123 (87)	18 (13)				
*Höchster Schulabschluss des Vaters*				0,1	2	0,995	0,01
– Ohne Abschluss/Hauptschule	41	36 (88)	5 (12)				
– Mittlere Reife	56	49 (87)	7 (13)				
– Abitur	133	116 (87)	17 (13)				
*Scheuch-Winkler-Index*				1,1	1	0,288	−0,07
– Niedriger/mittlerer SÖS	108	92 (85)	16 (15)				
– Hoher SÖS	162	145 (90)	17 (10)				
	***N***	**M (SD)**	**M (SD)**	**T**	**Df**	***p* (2-seitig)**	**d**
*Alter*	278	4,11 (1,0)	4,06 (1,2)	0,3	276	0,767	0,05
*Kindliche Verhaltensprobleme (CBCL)*							
– Externalisierend	275	11,8 (7,8)	13,2 (7,1)	−1,0	273	0,328	−0,18
– Internalisierend	275	8,8 (6,8)	9,8 (7,0)	−0,8	273	0,437	−0,14
*Psychische Belastung Mutter (DASS)*	275	23,4 (17,0)	26,3 (16,1)	−1,0	273	0,341	−0,18
*Psychische Belastung Vater (DASS)*	213	19,7 (14,1)	18,7 (16,0)	0,3	211	0,754	0,07

*CBCL* Child Behavior Checklist 1 ½‑5; *DASS* dt. Version der Depression-Anxiety-Stress-Scale; *** *p* ≤ 0,001, ** *p* ≤ 0,01, * *p* ≤ 0,05

### Vorhersage eines problematischen Konsums durch Variablen des Jugendalters

Die Ergebnisse der paarweisen Vergleiche mit den Variablen des Jugendalters (FU10) sind Tab. 3 zu entnehmen. Signifikante Unterschiede zwischen jungen Erwachsenen mit und ohne problematischen Konsum zeigten sich für die folgenden Variablen, die anschließend in die Berechnung des Klassifikationsbaums mit aufgenommen wurden: substanzunspezifischer Drogenkonsum (*p* = 0,006, φ = 0,17) sowie Cannabiskonsum (*p* = 0,006, φ = 0,17) im Jugendalter, das Ausmaß externalisierender Verhaltensauffälligkeiten in der Einschätzung der Mutter (*p* ≤ 0,001, *d* = −1,14) sowie im Selbsturteil (*p* = 0,016, *d* = −0,45), das Ausmaß internalisierender Verhaltensauffälligkeiten im Selbsturteil (*p* = 0,008, *d* = 0,38), das Ausmaß von Aufmerksamkeitsproblemen in der Einschätzung der Mutter (*p* ≤ 0,001, *d* = −0,66) und in Hinblick auf die Qualität der Eltern-Kind-Beziehung das Ausmaß an Kohäsion in der Beziehung zur Mutter (*p* = 0,050, *d* = 0,37), an mütterlicher Überprotektion (*p* = 0,040, *d* = −0,39) sowie an Konflikten mit der Mutter (*p* = 0,043, *d* = −0,38).

**Tab. 3:** Paarweiser Vergleich potenzieller Prädiktoren des Jugendalters (FU10) für einen problematischen Cannabiskonsum im jungen Erwachsenenalter (*N* = 278)

		Problematischer Cannabiskonsum					
		Nein	Ja				
Variablen des Jugendalters	***N***	***n* (%)**	***n* (%)**	**χ2**	**Df**	***p* (2-seitig)**	**φ**
*Bereits mind. eine Droge konsumiert*				7,4	1	0,006**	0,17
– Nein	213	191 (90)	22 (10)				
– Ja	43	32 (74)	11 (26)				
*Bereits mind. einmal Cannabis konsumiert*				7,6	1	0,006**	0,17
– Nein	224	200 (89)	24 (10)				
– Ja	32	23 (72)	9 (28)				
*Drogenkonsum im Freundeskreis*				2,6	2	0,271	0,10
– Nein, keiner	131	118 (90)	13 (10)				
– Ja, ein:e Freund:in	52	45 (87)	7 (13)				
– Ja, mind. 2 Freund:innen	73	60 (82)	13 (18)				
*Bereits Alkohol getrunken*				0,8	1	0,376	0,06
– Nein oder nur einmal probiert	150	133 (89)	17 (11)				
– Ja, mehrfach	106	90 (85)	16 (15)				
*Bereits mind. einmal geraucht*				1,2	1	0,270	0,07
– Nein	237	208 (88)	29 (14)				
– Ja	19	15 (79)	4 (21)				
*Aktueller Nikotinkonsum Mutter*				0,1	1	0,912	0,01
– Nein	186	162 (87)	24 (13)				
– Ja, gelegentlich oder täglich	67	58 (87)	9 (13)				
*Aktueller Nikotinkonsum Vater*				1,0	1	0,309	0,07
– Nein	158	141 (89)	17 (11)				
– Ja, gelegentlich oder täglich	78	66 (85)	12 (15)				
*Scheuch-Winkler-Index*				0,1	1	0,758	0,02
– Niedriger/mittlerer SÖS	95	84 (88)	11 (12)				
– Hoher SÖS	155	135 (87)	20 (13)				
*Besuchte Schulform*				1,9	1	0,165	−0,09
– Gymnasium	145	130 (90)	15 (10)				
– Andere Schulform	111	93 (84)	18 (16)				
	***N***	**M (SD)**	**M (SD)**	**T**	**Df**	***p* (2-seitig)**	**d**
*Kindliche Verhaltensprobleme aus Muttersicht CBCL*							
– Externalisierend	236	6,2 (6,6)	14,4 (10,6)	−4,1	31,1	≤ 0,001***	−1,14
– Internalisierend	236	6,3 (6,1)	8,3 (6,7)	−1,6	232	0,108	−0,32
– Skala Aufmerksamkeitsprobleme	236	2,3 (2,9)	4,2 (2,8)	−3,3	234	≤ 0,001***	−0,66
*Kindliche Verhaltensprobleme im Selbsturteil YSR*							
– Externalisierend	256	10,0 (6,2)	12,9 (8,4)	−2,4	254	0,016*	−0,45
– Internalisierend	256	10,2 (7,8)	7,4 (5,1)	2,8	57,8	0,008**	0,38
– Skala Aufmerksamkeitsprobleme	256	4,2 (2,9)	4,8 (2,6)	−1,1	254	0,256	−0,21
*Ausmaß Alkoholkonsum Mutter (LAST)*	253	0,4 (0,8)	0,4 (0,8)	0,0	251	0,973	0,01
*Ausmaß Alkoholkonsum Vater (LAST)*	182	0,7 (1,1)	0,6 (1,0)	0,3	180	0,794	0,01
*Psychische Belastung Mutter (DASS)*	234	20,0 (17,2)	19,1 (13,1)	0,3	232	0,786	0,05
*Psychische Belastung Vater (DASS)*	191	16,3 (12,7)	17,7 (18,4)	−0,4	25,0	0,714	−0,11
*Mutter-Kind-Beziehung (EBF-KJ)*							
– Kohäsion	256	14,1 (3,6)	12,8 (3,6)	2,0	254	0,050*	0,37
– Konflikte	256	7,1 (2,7)	8,2 (2,7)	−2,0	254	0,043*	−0,38
– Ablehnung	256	0,8 (1,8)	1,0 (1,4)	−0,6	254	0,523	−0,12
– Überprotektion	256	7,5 (3,2)	8,8 (3,3)	−2,1	254	0,040*	−0,39
*Vater-Kind-Beziehung (EBF-KJ)*							
– Kohäsion	243	11,8 (4,3)	11,6 (3,7)	0,3	241	0,770	0,06
– Konflikte	243	6,0 (2,8)	6,9 (2,8)	−1,7	241	0,091^#^	−0,33
– Ablehnung	243	0,9 (2,1)	0,9 (1,8)	−0,0	241	0,993	−0,00
– Überprotektion	243	5,4 (3,3)	6,3 (3,9)	−1,3	241	0,188	−0,26

*CBCL* Child Behavior Checklist 4–18; *DASS* dt. Version der Depression-Anxiety-Stress-Scale; *EBF-KJ* Elternbildfragebogen für Kinder und Jugendliche; *LAST* Lübecker Alkohol Screening Test; *YSR* Youth Self Report 11–18; *** *p* ≤ 0,001, ** *p* ≤ 0,01, * *p* ≤ 0,05, ^#^ *p* ≤ 0,10

Bei der Berechnung des Klassifikationsbaums wurden lediglich externalisierende Verhaltensauffälligkeiten aus Sicht der Mutter (CBCL_ext_) im ersten (*F* = 33,2; *df* = 1,234; *p* ≤ 0,001), zweiten (*F* = 17,1; *df* = 1,221; *p* ≤ 0,001) und dritten (*F* = 9,6; *df* = 1,221; *p* = 0,022) Schritt als signifikante Trennvariable identifiziert. Die Werte der vier durch die Analyse identifizierten Gruppen sind in Tab. 4 dargestellt. Der Anteil der jungen Erwachsenen mit problematischem Konsum stieg mit zunehmendem Ausmaß externalisierender Verhaltensprobleme im Jugendalter von 5,1 % bis 46,2 % deutlich an.

**Tab. 4:** Ergebnis des Klassifikationsbaums zur Vorhersage eines problematischen Cannabiskonsums im jungen Erwachsenenalter

	Junges Erwachsenenalter			
	Kein problematischer Konsum		Problematischer Cannabiskonsum	
Trennvariablen im Jugendalter	*n*	*%*	*n*	*%*
CBCL_ext_ > 21,48^a^	7	53,8	6	46,2
CBCL_ext_ = 17,86–21,47^b^	4	57,1	3	42,9
CBCL_ext_ = 6,72–17,85^c^	65	80,2	16	19,3
CBCL_ext_ < 6,71^d^	168	94,9	9	5,1

*CBCL*_*ext*_ Child Behavior Checklist 4–18 Jahre – Rohwert Syndromskala externalisierende Verhaltensprobleme; ^a^ T_♂_-Wert ≥ 67 (Jungen) bzw. T_♀_-Wert ≥ 72 (Mädchen); ^b^ T_♂_-Wert = 64–66 bzw. T_♀_-Wert = 69–71; ^c^ T_♂_-Wert = 52–63 bzw. T_♀_-Wert = 56–68; ^d^ T_♂_-Wert ≤ 52 bzw. T_♀_-Wert ≤ 54

## Diskussion

### Prävalenzen

In der aktuellen Studie lagen die Prävalenzraten für Cannabiskonsum im jungen Erwachsenenalter mit einer LZP von 57,6 %, einer 12‑M.-P. von 35,3 % und einer 30‑T.-P. von 20,1 % etwas höher als in der jüngsten repräsentativen Befragung der BZgA (LZP: 50,8 %; 12‑M.-P.: 25,0 %; 30‑T.-P.: 12,0 %; [10]). Eine mögliche Erklärung hierfür könnte der hohe Anteil Studierender in der aktuellen Stichprobe sein. In einer Studie aus dem Jahr 2012 betrug die LZP für Cannabiskonsum unter deutschen Studierenden 59,8 % und die 12‑M.-P. 37,5 % [38]. Ein problematischer Konsum wurde in der aktuellen Studie bei 12,2 % identifiziert. In einer retrospektiven Befragung junger Erwachsener, die im letzten Jahr Cannabis konsumiert hatten, wiesen 29,7 % einen riskanten Konsum auf [13]. Die entsprechend umgerechnete Prävalenzrate für einen problematischen Konsum unter den Cannabiskonsument:innen beträgt in der aktuellen Studie 34,7 % (34 von 98). Demnach weist etwa jede:r dritte junge Konsument:in einen problematischen Konsum auf.

#### Geschlechtsunterschiede.

Wie erwartet konsumierten männliche junge Erwachsene signifikant häufiger Cannabis als weibliche [9, 10, 13] mit einem 3,30-fach höheren Risiko für einen problematischen Konsum. Präventionsprogramme sollten sich daher besonders an junge Männer richten, dabei jedoch berücksichtigen, dass weibliches Konsumverhalten durch suchtpräventive Maßnahmen stärker beeinflusst wird [39].

#### Einfluss der COVID-19-Pandemie.

Drei Viertel der Befragten gaben keine Veränderung ihres Cannabiskonsums während der Pandemie an, was auf einen eher geringen Einfluss schließen lässt. Vorherige Studien fanden dagegen einen Anstieg des Cannabiskonsums während der Pandemie [40, 41]. In einer Studie stand dieser jedoch vor allem mit dem Verlust des Arbeitsplatzes in Zusammenhang [41]. In der aktuellen Studie waren 58 % der Konsument:innen Studierende, was den geringeren Einfluss erklären könnte.

### Entwicklung des Cannabiskonsums vom Jugend- zum jungen Erwachsenenalter

Zwei Drittel derjenigen, die im Jugendalter bereits Cannabis konsumiert hatten, taten dies auch als junge Erwachsene. Knapp ein Drittel wies einen problematischen Konsum auf. Im Vergleich zu den jungen Erwachsenen, die im Jugendalter noch kein Cannabis konsumiert hatten, war das Risiko für einen problematischen Konsum um das 2,63-Fache erhöht. Vor dem Hintergrund der gesundheitlichen Risiken eines frühen und problematischen [5–7] Cannabiskonsums ist dieses Ergebnis alarmierend. Präventionsmaßnahmen sollten frühzeitig ansetzen, um einem frühen Beginn und damit auch einem problematischen Konsum vorzubeugen.

### Risiko- und Schutzfaktoren des Kindesalters

Abgesehen vom biologischen Geschlecht zeigte sich bei der Variablenselektion lediglich hinsichtlich des Nettohaushaltseinkommens der Familie ein signifikanter Unterschied zwischen jungen Erwachsenen mit und ohne problematischen Konsum. Bei der anschließenden Berechnung des Klassifikationsbaums mit diesen beiden Variablen erwies sich ausschließlich das Geschlecht als signifikanter Prädiktor. In der Mannheimer Risikokinderstudie erwiesen sich hingegen ein gestörtes Sozial- und oppositionelles Verhalten in der Kindheit unabhängig vom Geschlecht der Kinder als Risikofaktoren für einen späteren problematischen Konsum [16]. Eine mögliche Erklärung dafür, warum sich das Ausmaß externalisierender Auffälligkeiten im Kindesalter in der aktuellen Studie als nicht signifikant erwies, könnte sein, dass es sich bei der aktuellen Studie um eine Präventionsstudie handelt, weshalb die Kinder insgesamt weniger verhaltensauffällig waren. Darüber hinaus wurde das Ausmaß der kindlichen Verhaltensauffälligkeiten für die Vorhersage in der Mannheimer Studie über drei Messzeitpunkte gemittelt (im Alter von 4,5, 8 und 11 Jahren), während in der vorliegenden Studie die Prädiktoren des Kindes- und Jugendalters unabhängig voneinander betrachtet wurden. Metaanalytische Befunde zeigen, dass (externalisierende) Verhaltensprobleme im Kindesalter das Risiko für den Konsum von Cannabis im jungen Erwachsenenalter nur dann erhöhen, wenn sie bis ins Jugendalter persistieren oder erst im Jugendalter beginnen [42].

### Risiko- und Schutzfaktoren des Jugendalters

Übereinstimmend mit früheren Befunden ergaben sich bei der Variablenselektion im Jugendalter zusätzlich zum biologischen Geschlecht für die folgenden Faktoren signifikante Unterschiede zwischen jungen Erwachsenen mit und ohne problematischen Cannabiskonsum: ein substanzunspezifischer Drogen- sowie Cannabiskonsum [13, 19, 20], stärker ausgeprägte externalisierende Verhaltensauffälligkeiten im Selbst- und Mutterbericht [17], stärkere Aufmerksamkeitsprobleme im Mutterbericht [16], niedrigere Kohäsion in der Beziehung zur Mutter, mütterliche Überprotektion und häufigere Konflikte mit der Mutter [18, 23]. Bei der Vorhersage des problematischen Cannabiskonsums mithilfe des Klassifikationsbaums erwies sich lediglich das Ausmaß externalisierender Verhaltensprobleme aus Muttersicht als signifikanter Risikofaktor.

Dieses Ergebnis stimmt mit zahlreichen früheren Studienergebnissen überein [15]. In einer US-amerikanischen Längsschnittstudie, in der vergleichbare Risikofaktoren untersucht wurden, erwiesen sich ebenfalls lediglich externalisierende Verhaltensprobleme sowie zusätzlich ein Substanzkonsum im jungen Erwachsenenalter (25–27 Jahre) als Prädiktoren für einen Missbrauch von Cannabis im späteren Erwachsenenalter (32–34 Jahre; [43]). Die aktuellen Ergebnisse bestätigen außerdem den Befund, dass sich Geschlechtsunterschiede nicht länger als signifikant erweisen, wenn in den Analysen andere relevante Einflussfaktoren berücksichtigt werden [22, 43, 44].

Eine Reihe früherer Studien ergab weiterhin, dass der Zusammenhang zwischen externalisierenden Verhaltensproblemen im Jugendalter und einem späteren Cannabisgebrauch [45] sowie CUD im jungen Erwachsenenalter [44] teilweise durch einen früheren Konsum von Zigaretten und Alkohol moderiert bzw. mediiert wird. Dieser Befund wurde in der vorliegenden Studie nicht bestätigt. Allerdings wurde der Konsum von Alkohol und Nikotin im Jugendalter auch nicht in die Vorhersage mit aufgenommen, da sich bei der Variablenselektion kein signifikanter Unterschied zwischen jungen Erwachsenen mit und ohne problematischen Konsum zeigte. Eine denkbare Erklärung hierfür könnte sein, dass bei der FU10-Befragung lediglich 41 % der Jugendlichen bereits mehrfach Alkohol getrunken und nur 7 % schon einmal geraucht hatten. Möglicherweise waren die Jugendlichen zum FU10 mit im Mittel 14,1 Jahren zu jung, um diese Risikofaktoren angemessen untersuchen zu können.

Ebenso wurden der elterliche Substanzkonsum, die elterliche psychische Belastung und der SÖS nicht in das Vorhersagemodell mit aufgenommen. Die nichtsignifikanten Unterschiede bei diesen Variablen könnten darauf zurückzuführen sein, dass in der aktuellen Studie eine insgesamt wenig belastete Stichprobe untersucht wurde und Familien mit einem niedrigen SÖS unterrepräsentiert waren, weshalb eine Replikation der Analysen an anderen Stichproben zu empfehlen ist.

### Stärken und Limitationen der Studie

Die größten Stärken der Studie sind das längsschnittliche Design des Projekts „Zukunft Familie“ und die prospektive Erhebung diverser kindlicher und familiärer Faktoren über 18 Jahre. In diesem Zusammenhang weitere Stärken sind die wiederholte Erfassung des Cannabiskonsums im Jugend- und jungen Erwachsenalter, die Erhebung jugendlicher Verhaltensprobleme im Selbst- und Mutterbericht sowie die Berücksichtigung mütterlicher und väterlicher Risikofaktoren.

Folgende Limitationen sind jedoch ebenfalls zu berücksichtigen:

1. Die Generalisierbarkeit ist eingeschränkt aufgrund der begrenzten Stichprobengröße, der Unterrepräsentation von Familien mit einem niedrigen SÖS sowie der insgesamt wenig belasteten Stichprobe.
2. Für den CUDIT‑R liegen bislang keine deutschen Cut-off-Werte vor. Dadurch könnte die Häufigkeit eines problematischen Konsums über- oder unterschätzt worden sein.
3. Bedingt durch das junge Alter zum FU10 fielen die Prävalenzraten des Substanzkonsums im Jugendalter sehr gering aus, sodass diese Variablen als Prädiktoren nur bedingt und wenig differenziert untersucht werden konnten.
4. Bei der Selektion der Variablen des Kindes- und Jugendalters wurden jeweils mehrere zusammenhängende Merkmale betrachtet, sodass mit einer Alphafehlerkumulierung gerechnet werden muss.
5. Die Ergebnisse des Klassifikationsbaums sollten vorsichtig interpretiert werden, da die Stichprobengrößen der Endknoten teilweise sehr klein waren.
6. Cannabiskonsum als sensibles Thema und die Illegalität von Cannabis während der Erhebungszeiträume könnten zu Antworten im Sinne der sozialen Erwünschtheit geführt haben.
7. Ein Großteil der FU18-Befragungen fand während der COVID-19-Pandemie statt, was die Reliabilität der Daten beeinträchtigt haben könnte, obwohl die meisten jungen Erwachsenen keine Veränderung ihres Konsums angaben.

### Implikationen für die Praxis

Die aktuellen Ergebnisse betonen die hohe Relevanz des Schutzes von Jugendlichen und jungen Erwachsenen im Zusammenhang mit der Cannabislegalisierung. Um Kinder und Jugendliche vor den negativen Einflüssen von Cannabis zu schützen, bedarf es laut einer Stellungnahme der Suchtkommission der kinder- und jugendpsychiatrischen und medizinischen Fachgesellschaften und Verbände aus dem Jahr 2021 statt einer Legalisierung einer strikten Angebotsreduzierung, da sich verhaltenspräventive Maßnahmen zur Suchtprävention für Kinder und Jugendliche in der Vergangenheit als kaum wirksam erwiesen hätten [4]. Unterstützt wird diese Forderung durch Studienergebnisse, die zeigen, dass beispielsweise US-amerikanische junge Erwachsene nach einer Legalisierung von Cannabis dieses signifikant häufiger konsumierten als vor der Legalisierung 10 Jahre zuvor [46]. Um nach der Legalisierung die Erreichbarkeit von Cannabis für Kinder und Jugendliche zu erschweren, scheint folglich eine strikte Einhaltung und Kontrolle der im CanG vorgeschriebenen Mindestaltersgrenze von 18 Jahren sowie des öffentlichen Konsumverbots in den vorgesehenen Schutzzonen um öffentliche Einrichtungen, wie Schulen und Spielplätze, dringend notwendig. Da die praktische Umsetzung solcher Konsumverbote jedoch immer wieder angezweifelt wird [2–4], sollte zusätzlich eine flächendeckende präventive Aufklärungs- und Bildungsoffensive zu Cannabis an Schulen eingeführt werden [47, 48]. Die Ergebnisse der aktuellen Studie legen nahe, präventive Angebote speziell an Jugendliche mit externalisierenden Verhaltensauffälligkeiten zu richten. Hierbei könnte es ein Ziel sein, über Informationen zu positiven und negativen Konsumfolgen, wie dem Einfluss auf das unausgereifte Gehirn, sowie weniger gesundheitsschädlichen Konsumformen das Risikobewusstsein und damit reflektierte Konsumentscheidungen zu fördern sowie die Widerstandkraft gegen einen verfrühten Substanzkonsum zu erhöhen [4, 47].

Gemäß den aktuellen Ergebnissen sollte bei der Planung und dem Ausbau präventiver Angebote auch die Prävention externalisierender Verhaltensauffälligkeiten berücksichtigt werden. Ergänzend zu kindzentrierten Angeboten könnten hier familienzentrierte Angebote, wie Elterntrainings, zum Einsatz kommen [49]. In der aktuellen Studie erwies sich die elterliche Teilnahme am präventiven Elterntraining „Triple P“ im Kindesalter nicht als Schutzfaktor für einen problematischen Cannabiskonsum im jungen Erwachsenenalter, jedoch gilt dessen Wirksamkeit zur Prävention kindlicher externalisierender Verhaltensprobleme international als bestätigt [50].

### Implikationen für die Forschung

Bereits im Vorfeld der Legalisierung wurde immer wieder die dringende Notwendigkeit einer wissenschaftlichen Begleitforschung und deren Finanzierung betont [47, 48]. Das CanG sieht 18 Monate nach Inkrafttreten eine erste Evaluation u. a. der Auswirkungen auf den Konsum von Kindern und Jugendlichen vor sowie eine umfassende, abschließende Evaluation nach 4 Jahren. Mit Blick auf die aktuellen Ergebnisse sollten in diesem Rahmen neben den Prävalenzraten auch der Konsumbeginn, das Vorliegen eines problematischen Konsums oder sogar einer CUD sowie relevante Risiko- und Schutzfaktoren systematisch mit erhoben und ausgewertet werden. Darüber hinaus scheint es dringend geboten, die mittel- und langfristigen Auswirkungen der Legalisierung im Rahmen groß angelegter Längsschnittstudien mit repräsentativen Stichproben zu untersuchen. Der Studienzeitraum sollte dabei idealerweise mindestens das frühe Jugend- bis späte junge Erwachsenenalter umfassen, um die Entwicklung der Prävalenzraten sowie eines problematischen Konsums zuverlässig abbilden und Ansatzpunkte für zielgruppenspezifische Präventions- und Behandlungsangebote identifizieren zu können. Dabei sollten auch weitere potenzielle Risikofaktoren berücksichtigt werden, wie Trennung der Eltern, Tod eines Elternteils [50] und elterlicher Cannabiskonsum [21]. Weiterer Forschungsbedarf besteht auch in dem Bereich Muster und Motive des Konsumverhaltens [51].

## Fazit

Die aktuellen Ergebnisse bestätigen, dass der Konsum von Cannabis ebenso wie ein problematischer Konsum im jungen Erwachsenenalter alles andere als eine Seltenheit sind. Sie betonen damit nicht nur die Notwendigkeit der strikten Umsetzung verhältnispräventiver Maßnahmen und den Ausbau verhaltenspräventiver Angebote unter Einbezug der bisherigen Erkenntnisse der Risiko- und Schutzfaktorenforschung, sondern auch einer systematischen wissenschaftlichen Begleitforschung zu den Auswirkungen der Legalisierung. Sowohl hinsichtlich der Entwicklung der Prävalenzraten und der Konsumstabilität als auch hinsichtlich der Effektivität präventiver Maßnahmen sind systematische Längsschnittstudien erforderlich. Diese sollten mindestens mehrere Jahre umfassen, um belastbare Aussagen zu den Auswirkungen der Legalisierung und den Erfolg präventiver Bemühungen treffen zu können.

## Supplementary Information

Im Onlinematerial befinden sich weitere Tabellen mit soziodemographischen Daten der 18-Jahres-Follow-up Stichprobe der jungen Erwachsenen (Tabelle Z1), den soziodemographischen Daten der Familien der Längsschnittstudie „Zukunft Familie” zu Prä, FU10 und FU18 (Tabelle Z2) sowie eine Übersicht über die geschlechtsspezifischen Prävalenzraten für Cannabiskonsum und einen problematischen Konsum bei den jungen Erwachsenen (Tabelle Z3).

## References

[1] Jayawardhana J, Hou J, Freeman P et al (2024) Association of state cannabis legalization with Cannabis Use Disorder and cannabis poisoning. JAMA Psychiatry. 10.1001/jamapsychiatry.2024.4145 10.1001/jamapsychiatry.2024.4145 PMC11883495 39714863

[2] Bundesärztekammer (2023) Stellungnahme zum Entwurf eines Gesetzes der Bundesregierung zum kontrollierten Umgang mit Cannabis und zur Änderung weiterer Vorschriften (Cannabisgesetz – CanG) (BT-Drs. 20/8704) vom 06.11.2023. https://kripoz.de/wp-content/uploads/2023/11/20_14_0154-11-_Bundesaerztekammer_Cannabis-data.pdf. Zugegriffen: 26. Jan. 2025

[3] Bundespsychotherapeutenkammer (2023) Stellungnahme zum Gesetz zum kontrollierten Umgang mit Cannabis und zur Änderung weiterer Vorschriften (CanG) vom 19.10.2023. https://kripoz.de/wp-content/uploads/2023/11/20_14_0154-1-_Bundespsychotherapeutenkammer-BPtK-_Cannabis_nicht-barrierefrei-data.pdf. Zugegriffen: 6. Nov. 2024

[4] Suchtkommission der kinder- und jugendpsychiatrischen und kinder- und jugendmedizinischen Fachgesellschaften und Verbände in Deutschland (2021) Gesundheitliche Risiken einer Cannabislegalisierung für Kinder und Jugendliche. https://www.dgkjp.de/wp-content/uploads/Punchpaper-Cannabislegalisierung_fin.pdf. Zugegriffen: 15. Jan. 2025

[5] Meier MH, Caspi A, Ambler A et al (2012) Persistent cannabis users show neurological decline from childhood to midlife. Proc Natl Acad Sci USA 109:2657–2664. 10.1073/pnas.1206820109 10.1073/pnas.1206820109 PMC3479587 22927402

[6] Hjorthøj C, Compton W, Starzer M et al (2023) Association between cannabis use disorder and schizophrenia stronger in young males than in females. Psychol Med 53:7322–7328. 10.1017/S0033291723000880 37140715 10.1017/S0033291723000880 PMC10719679

[7] Gobbi G, Atkin T, Zytynski T et al (2019) Association of cannabis use in adolescence and risk of depression, anxiety, and suicidality in young adulthood. A systematic review and meta-analysis. JAMA Psychiatry 76:426–434. 10.1001/jamapsychiatry.2018.4500 30758486 10.1001/jamapsychiatry.2018.4500 PMC6450286

[8] Chan O, Daudi A, Ji D et al (2024) Cannabis use during adolescence and young adulthood and academic achievement. A systematic review and meta-analysis. JAMA Pediatr 178:1280–1289. 10.1001/jamapediatrics.2024.3674 39374005 10.1001/jamapediatrics.2024.3674 PMC11459363

[9] Rauschert C, Möckl J, Seitz NN, Wilms N, Olderbak S, Kraus L (2022) Konsum psychoaktiver Substanzen in Deutschland. Dtsch Ärztebl 119:527–534. 10.3238/arztebl.m2022.0244 10.3238/arztebl.m2022.0244 PMC9677535 35791270

[10] Orth B, Merkel C (2022) Der Substanzkonsum Jugendlicher und junger Erwachsener in Deutschland. Ergebnisse des Alkoholsurveys 2021 zu Alkohol, Rauchen, Cannabis und Trends. BZgA-Forschungsbericht. Bundeszentrale für gesundheitliche Aufklärung 10.17623/BZGA:Q3-ALKSY21-DE-1.0

[11] Arnaud N, Wartberg L, Simon-Kutscher K, Thomasius R (2024) Prevalence of substance use disorders and associations with mindfulness, impulsive personality traits and psychopathological symptoms in a representative sample of adolescents in Germany. Eur Child Adolesc Psychiatry 33:451–465. 10.1007/s00787-023-02173-0 (IMAC-Mind Consortium) 36853515 10.1007/s00787-023-02173-0 PMC9972301

[12] Leung L, Chan GCK, Hides L, Hall WD (2020) What is the prevalence and risk of cannabis use disorders among people who use cannabis? A systematic review and meta-analysis. Addict Behav. 10.1016/j.addbeh.2020.106479 32485547 10.1016/j.addbeh.2020.106479

[13] Seidel AK, Morgenstern M, Hanewinkel R (2020) Risikofaktoren für einen riskanten Cannabiskonsum. Nervenarzt 91:1040–1046. 10.1007/s00115-020-00930-z 32488414 10.1007/s00115-020-00930-z

[14] Robinson T, Ali MU, Easterbrook B et al (2022) Identifying risk-thresholds for the association between frequency of cannabis use and development of cannabis use disorder: a systematic review and meta-analysis. Drug Alcohol Depend. 10.1016/j.drugalcdep.2022.109582 35932748 10.1016/j.drugalcdep.2022.109582

[15] Solmi M, Dragioti E, Croatto G et al (2021) Risk and protective factors for cannabis, cocaine, and opioid use disorders: an umbrella review of meta-analyses of observational studies. Neurosci Biobehav Rev 126:243–251. 10.1016/j.neubiorev.2021.03.014 33737104 10.1016/j.neubiorev.2021.03.014

[16] Zohsel K, Baldus C, Schmidt MH et al (2016) Predicting later problematic cannabis use from psychopathological symptoms during childhood and adolescence: Results of a 25-year longitudinal study. Drug Alcohol Depend 163:251–255. 10.1016/j.drugalcdep.2016.04.012 27114206 10.1016/j.drugalcdep.2016.04.012

[17] Haller AC, Klasen F, Petermann F et al (2016) Langzeitfolgen externalisierender Verhaltensauffälligkeiten. Kindh Entwickl 25:31–40. 10.1026/0942-5403/a000186

[18] Loxley W, Toumbourou J, Stockwell T et al (2004) The prevention of substance use, risk and harm in Australia: a review of the evidence. National Drug Research Institute, Commonwealth of Australia. http://www.health.gov.au/internet/main/publishing.nsf/Content/health-pubhlth-publicat-document-mono_prevention-cnt.htm. Zugegriffen: 1. Aug. 2024

[19] Hohm E, Blomeyer D, Schmidt MH, Esser G, Laucht M (2007) Jugendliche, die frühzeitig rauchen und trinken-eine Risikogruppe? Z Psychiatr Psychol Psychother 55:155–165. 10.1024/1661-4747.55.3.155

[20] Chen CY, Storr CL, Anthony JC (2009) Early-onset drug use and risk for drug dependence problems. Addict Behav 34:319–322. 10.1016/j.addbeh.2008.10.021 19022584 10.1016/j.addbeh.2008.10.021 PMC2677076

[21] Swift W, Coffey C, Carlin JB, Degenhardt L, Patton GC (2008) Adolescent cannabis users at 24 years: trajectories to regular weekly use and dependence in young adulthood. Addiction 103:1361–1370. 10.1111/j.1360-0443.2008.02246.x 18855826 10.1111/j.1360-0443.2008.02246.x

[22] Brook JS, Brook DW, Arencibia-Mireles O, Richter L, Whiteman M (2001) Risk factors for adolescent marijuana use across cultures and across time. J Genet Psychol 162:357–374. 10.1080/00221320109597489 11678369 10.1080/00221320109597489

[23] Esser G, Ihle W (2008) Abhängigkeit von legalen und illegalen psychotropen Substanzen. Kindh Entwickl 17:1–4. 10.1026/0942-5403.17.1.1

[24] von Sydow K, Lieb R, Pfister H, Höfler M, Wittchen HU (2002) What predicts incident use of cannabis and progression to abuse and dependence? A 4-year prospective examination of risk factors in a community sample of adolescents and young adults. Drug Alcohol Depend 68:49–64. 10.1016/S0376-8716(02)00102-3 12167552 10.1016/s0376-8716(02)00102-3

[25] Sanders MR, Kirby JN, Tellegen CL, Day JJ (2014) The triple P‑positive parenting program: a systematic review and meta-analysis of a multi-level system of parenting support. Clin Psychol Rev 34:337–357. 10.1016/j.cpr.2014.04.003 24842549 10.1016/j.cpr.2014.04.003

[26] Hahlweg K, Schulz W (2018) Universelle Prävention kindlicher Verhaltensstörungen durch Elterntrainings. Z Klin Psychol Psychother 47:1–15. 10.1026/1616-3443/a000462

[27] Adamson SJ, Kay-Lambkin FJ, Baker AL et al (2010) An improved brief measure of cannabis misuse: the Cannabis Use Disorders Identification Test-Revised (CUDIT-R). Drug Alcohol Depend 110:137–143. 10.1016/j.drugalcdep.2010.02.017 20347232 10.1016/j.drugalcdep.2010.02.017

[28] Bonn-Miller MO, Heinz AJ, Smith EV, Bruno R, Adamson S (2016) Preliminary development of a brief cannabis use disorder screening tool: the cannabis use disorder identification test short-form. Cannabis Cannabinoid Res 1:252–261. 10.1089/can.2016.0022 28861497 10.1089/can.2016.0022 PMC5531365

[29] Winkler J, Stolzenberg H (1998) Der Sozialschichtindex im Bundes-Gesundheitssurvey. Gesundheitswesen 61:178–183 10726418

[30] Lange M, Kamtsiuris P, Lange C, Schaffrath Rosario A, Stolzenberg H, Lampert T (2007) Messung soziodemographischer Merkmale im Kinder- und Jugendgesundheitssurvey (KiGGS) und ihr Bedeutung am Beispiel der Einschätzung des allgemeinen Gesundheitszustands. Bundesgesundheitsbl Gesundheitsforsch Gesundheitsschutz 50:578–589. 10.1007/s00103-007-0219-5 10.1007/s00103-007-0219-5 17514442

[31] Arbeitsgruppe Deutsche Child Behavior Checklist (2000) Elternfragebogen für Klein- und Vorschulkinder (CBCL 1 ½–5). Hogrefe, Göttingen

[32] Döpfner M, Plück J, Kinnen C et al. (2014) Deutsche Schulalter-Formen der Child Behavior Checklist von Thomas M. Achenbach – Elternfragebogen über das Verhalten von Kindern und Jugendlichen (CBCL/6–18R), Lehrerfragebogen über das Verhalten von Kindern und Jugendlichen (TRF/6–18R), Fragebogen für Jugendliche (YSR/11–18R). Hogrefe, Göttingen.

[33] Köppe E (2001) Glückliche Eltern – liebe Kinder? Auswirkungen von Partnerschaft und psychischer Symptomatik der Eltern auf das Verhalten ihrer Kinder. Technische Universität Carolo-Wilhelmina zu Braunschweig (Unveröffentlichte Dissertation)

[34] Rumpf HJ, Hapke U, John U (2001) LAST. Lübecker Alkoholabhängigkeits- und -missbrauchs-Screening-Test (Testmanual). Hogrefe, Göttingen

[35] Titze K, Lehmkuhl U (2010) Elternbildfragebogen für Kinder und Jugendliche (EBF-KJ). Manual. Hogrefe, Göttingen

[36] Rokach L, Maimon O (2010) Classification trees. In: Maimon O, Rokach L (Hrsg) Data mining and knowledge discovery handbook. Springer, Heidelberg, S 149–174

[37] IBM Corp (2022) IBM SPSS Statistics for Windows, Version 28.0. IBM Corp

[38] Pauly A, Klein M (2012) Cannabiskonsum im Studium. Sucht 58:127–135. 10.1024/0939-5911.a000172

[39] Bühler A, Kröger C (2006) Expertise zur Prävention des Substanzmissbrauchs. Bundeszentrale für gesundheitliche Aufklärung. https://shop.bzga.de/pdf/60629000.pdf. Zugegriffen: 1. Aug. 2024

[40] Bartel SJ, Sherry SB, Stewart SH (2020) Self-isolation: a significant contributor to cannabis use during the COVID-19 pandemic. Subst Abus 41:409–412. 10.1080/08897077.2020.1823550 33044893 10.1080/08897077.2020.1823550

[41] Pocuca N, London-Nadeau K, Geoffroy MC et al (2022) Changes in emerging adults’ alcohol and cannabis use from before to during the COVID-19 pandemic: evidence from a prospective birth cohort. Psychol Addict Behav 36:786–797. 10.1037/adb0000826 35201807 10.1037/adb0000826

[42] Bevilacqua L, Hale D, Barker ED, Viner R (2018) Conduct problems trajectories and psychosocial outcomes: a systematic review and meta-analysis. Eur Child Adolesc Psychiatry 27:1239–1260. 10.1007/s00787-017-1053-4 28983792 10.1007/s00787-017-1053-4

[43] Lansford JE, Goulter N, Godwin J et al (2023) Predictors of problematic adult alcohol, cannabis, and other substance use: a longitudinal study of two samples. Dev Psychopathol 35:2028–2043. 10.1017/S0954579422000670 35957585 10.1017/S0954579422000670 PMC9922340

[44] Hayatbakhsh MR, McGee TR, Bor W, Najman JM, Jamrozik K, Mamun AA (2008) Child and adolescent externalizing behavior and cannabis use disorders in early adulthood: an Australian prospective birth cohort study. Addict Behav 33:422–438. 10.1016/j.addbeh.2007.10.004 17996381 10.1016/j.addbeh.2007.10.004

[45] Korhonen T, Prince von Leeuwen A, Reijneveld SA, Ormel J, Verhulst FC, Huizink AC (2010) Externalizing behavior problems and cigarette smoking as predictors of cannabis use: the TRAILS Study. J Am Acad Child Adolesc Psychiatry 49:61–69. 10.1016/j.jaac.2009.09.001 20215927 10.1097/00004583-201001000-00010

[46] Stormshak EA, Caruthers AS, Gau JM, Winter C (2019) The impact of recreational marijuana legalization on rates of use and behavior: a 10-year comparison of two cohorts from high school to young adulthood. Psychol Addict Behav 33:595–602. 10.1037/adb0000508 31424245 10.1037/adb0000508 PMC6856398

[47] Branchenverband Cannabiswirtschaft e. V. (2022) Eckpunktepapier Genussmittelregulierung. Auf dem Weg zu einer Deutschen Cannabis Agenda. https://start.cannabiswirtschaft.de/wp-content/uploads/2022/02/ELEMENTE_20_Eckpunktepapier_Genussmittelregulierung_BvCW.pdf. Zugegriffen: 1. Aug. 2024

[48] Deutsche Gesellschaft für Psychiatrie und Psychotherapie, Psychosomatik und Nervenheilkunde e. V. (2022) Cannabis-Legalisierung: Prävention und Jugendschutz sind nicht verhandelbar. Positionspapier. https://www.dgppn.de/_Resources/Persistent/d1c7d0a1abdcbed257d3ef1d4e21418d29987016/2022-03-29_DGPPN-Positionspapier_Cannabislegalisierung_FIN.pdf. Zugegriffen: 1. Aug. 2024

[49] Bühler A, Thrul J, Gomes de Matos E (2020) Expertise zur Suchtprävention 2020. Aktualisierte Neuauflage der „Expertise zur Suchtprävention 2013“. https://shop.bzga.de/pdf/60640052.pdf. Zugegriffen: 1. Aug. 2024

[50] Hoch E, Lieb R (2009) Substanzmissbrauch und -abhängigkeit. In: Schneider S, Margraf J (Hrsg) Lehrbuch der Verhaltenstherapie. Springer, Heidelberg, S 763–783

[51] Suhren E, Dewitz M, Bodemer N, Lohmann K (2021) Forschungsaktivitäten zu den Auswirkungen von COVID-19 auf den Substanzkonsum, die Entwicklung von Verhaltenssüchten sowie das Suchthilfesystem. Institut für Innovation und Technik (iit) in der VDI/VDE-IT. https://www.bundesgesundheitsministerium.de/fileadmin/Dateien/5_Publikationen/Drogen_und_Sucht/Berichte/Abschlussbericht/Corona_und_Sucht_Abschlussberichtpdf. Zugegriffen: 1. Aug. 2024

