# Supplementary material for: Development of cannabis use from adolescence to young adulthood and risk and protective factors for problematic use—results of a longitudinal study
Source: Bundesgesundheitsblatt Gesundheitsforschung Gesundheitsschutz. 2025 Apr 15;68(5):548–59. [Article in German] doi: 10.1007/s00103-025-04043-3 (PMC12075276; doi:10.1007/s00103-025-04043-3)
Supplement: Supplementary file 1 — Im Onlinematerial befinden sich weitere Tabellen mit soziodemographischen Daten der 18-Jahres-Follow-up Stichprobe der jungen Erwachsenen (Tabelle Z1), den soziodemographischen Daten der Familien der Längsschnittstudie „Zukunft Familie” zu Prä, FU10 und FU18 (Tabelle Z2) sowie eine Übersicht über die geschlechtsspezifischen Prävalenzraten für Cannabiskonsum und einen problematischen Konsum bei den jungen Erwachsenen (Tabelle Z3). [file 103_2025_4043_MOESM1_ESM.pdf]

Onlinematerial

Tabelle Z1. Soziodemographische Daten der 18-Jahres-Follow-up-Stichprobe der jungen Erwachsenen mit vollständigen Daten zum Cannabiskonsum (FU18; *N* = 278).

Charakteristika	*n (%)*
Biologisches Geschlecht	
- weiblich	140 (50)
- männlich	138 (50)
Aktuelle Wohnsituation	
- wohnhaft bei mind. einem Elternteil	86 (31)
- bereits ausgezogen	192 (69)
Aktuelle feste Partnerschaft	
- nein	151 (54)
- ja	127 (46)
Bereits eigene Kinder	
- nein	269 (3)
- ja	9 (97)
Schulabschluss junge Erwachsene (FU18)	
- ohne Abschluss/ Hauptschul- / Förderschulabschluss	35 (9)
- Mittlere Reife	44 (16)
- Abitur / (Fach-)Hochschulreife	209 (75)
Bereits eine abgeschlossene Berufsausbildung	
- nein	89 (32)
- ja	189 (68)
Aktuelle Tätigkeit	
- Studium an einer Universität, (Fach-)Hochschule	150 (54)
- Ausbildung / Lehre	36 (13)
- ausschließlich Berufstätigkeit	55 (20)
- arbeitssuchend	20 (7)
- andere	17 (6)

Tabelle Z2. Soziodemographische Daten der Familien der Längsschnittstudie „Zukunft Familie“ zu Prä, FU10 und FU18.

	Prä	FU10	FU18
Erhebungszeitraum (Jahr)	2001 – 2004	2011 – 2013	2020 – 2022
Charakteristika	*n* (%)	*n* (%)	*n* (%)
N (Familien)	477 (100)	361 (76)	316 (67)[1]
Biologisches Geschlecht			
- weiblich	224 (47)	166 (46)	153 (48)
- männlich	253 (53)	195 (54)	163 (52)
Migrationshintergrund (mind. eines Elternteils)			
- nein	375 (79)	291 (81)	257 (81)
- ja	102 (21)	70 (19)	59 (19)
Familienform			
- Zweielternfamilie	347 (73)	240 (66)	171 (54)
- Einelternfamilie[2]	129 (27)	110 (31)	143 (45)
- fehlende Werte	1 (0)	11 (3)	2 (1)
Sozioökonomischer Status			
- niedrig	30 (6)	12 (3)	5 (2)
- mittel	197 (41)	138 (38)	130 (41)
- hoch	230 (48)	196 (54)	151 (48)
- fehlende Werte	20 (4)	15 (4)	30 (9)
Triple P-Elterntraining			
- Interventionsgruppe	383 (80)	282 (78)	246 (78)
- Kontrollgruppe	94 (20)	79 (22)	70 (22)

	M	*SD*	*Min*	*Max*	*M*	*SD*	*Min*	*Max*	*M*	*SD*	*Min*	*Max*
Alter (Jahre)	4.2	1.0	2	7	14.1	1.2	11	17	22.3	1.7	19	26

Anmerkungen. [1]*N* = 6 Familien wurden nicht erneut erhoben, da sie die Einschlusskriterien nicht erfüllten; [2]aufgrund elterlicher Trennung, da Eltern nie eine Beziehung hatten oder aufgrund des Tods eines Elternteils.

Tabelle Z3. Vergleich der verschiedenen Prävalenzraten für allgemeinen Cannabiskonsum sowie problematischen Konsum zwischen männlichen und weiblichen jungen Erwachsenen.

	Gesamt	Männer	Frauen			
	n (%)	*n* (%)	*n* (%)	$\chi 2$	*p*	φ
Gesamtstichprobe	278 (100)	138 (49.6)	140 (50.4)			
Lebenszeitprävalenz	160 (57.6)	90 (65.2)	70 (50.0)	6.59	.005**	.154
12-Monatsprävalenz	98 (35.3)	62 (44.9)	36 (25.7)	11.24	≤ .001***	.201
30-Tage-Prävalenz	56 (20.1)	37 (26.8)	19 (13.6)	7.57	.003**	.165
Problem. Konsum[a]	34 (12.2)	26 (18.8)	8 (5.7)	11.16	≤ .001***	.200

Anmerkungen. [a] Summenwert CUDIT-R ≥ 10; *p* (1-seitig); *** $p \leq .001$, ** $p \leq .01$, * $p \leq .05$.
